# Chikungunya Outbreak, Singapore, 2008

**DOI:** 10.3201/eid1505.081390

**Published:** 2009-05

**Authors:** Yee S. Leo, Angela L.P. Chow, Li Kiang Tan, David C. Lye, Li Lin, Lee C. Ng

**Affiliations:** Tan Tock Seng Hospital, Singapore (Y.S. Leo, A.L.P. Chow, D.C. Lye, L. Lin); National Environment Agency, Singapore (L.K. Tan, L.C. Ng); **References**

**Keywords:** Vector-borne diseases, viruses, chikungunya, Aedes aegypti, Aedes albopictus, autochthonous, outbreak, isolation, Singapore, letter

**To the Editor:** Chikungunya virus, an arbovirus belonging to the family *Togaviridae*, genus *Alphavirus*, was first isolated in Tanzania in 1953 (*1*). The first outbreak in Asia was documented in Bangkok, Thailand, in 1958. Since then, outbreaks have been reported in Cambodia, Vietnam, Laos, Myanmar, Malaysia, the Philippines, and Indonesia (*2*). In Indonesia, a 1972 serosurvey suggested widespread distribution of chikungunya infection, and numerous outbreaks have reemerged since 2001 (*3*). Malaysia reported its first outbreak between December1998 and February 1999 and a reemergence in an isolated northwestern coastal town in 2006 (*4*).

In Singapore, although dengue fever has been endemic since the 1960s, the first chikungunya case was not reported until 2006. In 2007, 10 imported cases were reported to Singapore’s Ministry of Health (*5*). Notably, Taiwan reported a case involving a returning student from Singapore in November 2006, suggesting the possibility of autochthonous transmission in Singapore (*6*).

Located in tropical Southeast Asia, Singapore has remained vigilant in the surveillance of chikungunya. A 2002/2003 serosurvey on 531 healthy young adults showed only 2 (0.3%) persons with chikungunya antibodies (*7*). We describe an outbreak of autochthonous chikungunya transmission in Singapore and discuss removal of infectious human reservoirs from transmission areas as an outbreak control strategy.

On January 14, 2008, a local case of chikungunya infection was detected through the general practitioners’ laboratory-based surveillance system established by Singapore’s Environmental Health Institute in 2006. The Ministry of Health responded with a massive active surveillance exercise. A total of 2,626 people who resided or worked within a 150-m radius of the index case-patient’s address were screened for chikungunya infection by reverse transcription–PCR (RT-PCR), using primers adapted from Hasebe et al. (*5**,**8*). Persons with an acute febrile illness, signs or symptoms compatible with chikungunya fever (fever, joint pain, or rash), or those with positive RT-PCR results were referred to the Communicable Disease Centre at Tan Tock Seng Hospital (CDC/TTSH), the national infectious disease referral center in Singapore.

During the outbreak period from January 14 to February 21, 2008, chikungunya infection was confirmed for 13 patients (*5*). Of these, 10 acutely symptomatic patients (all men; median age 35 years, range 22–69 years) were isolated at CDC/TTSH until fever resolved and a negative chikungunya RT-PCR test result was obtained. During hospitalization, patients’ temperatures were monitored every 4 hours and daily chikungunya RT-PCR tests were performed. Viral load profiles were derived from an external standard curve generated by 10-fold serially diluted virus from a concentration of 10^8^ pfu/mL, using crossing-point values.

 The Table summarizes the presence of viremia and patients’ febrile status in relation to the day of illness. High levels of viremia were observed during the first 5 days of illness (median 119,126 pfu/mL, range 360–14,605,314 pfu/mL). Fever lasted a median of 5 days (range 3–10 days); viremia persisted up to day 9 of illness. Our findings concur with those of a European study, suggesting extremely high levels of viremia at the initial stage of chikungunya disease (*9*). Notably, 1 patient (patient 4), who was screened by the Ministry of Health, was observed to have a positive chikungunya RT-PCR test result 1 day before symptom onset. Fever resolution did not predict viral clearance. Of note, 30% of our patients had detectable viremia (376–8,523 pfu/mL), after fever had resolved. We are uncertain of the role of level of viremia in the transmission of chikungunya; more research is needed to address this pertinent public health question.

**Table:** Daily trend of fever and viremia in 10 hospitalized chikungunya patients, Singapore (www.cdc.gov/EID/content/15/5/836-T.htm)

Patient no.	Signs and symptoms*	Fever† and chikungunya test results (viral load‡), by day of fever											
		–1	0	1	2	3	4	5	6	7	8	9	10
1	F, A, B, D					NA + (442.7)	37.7§ + (3.5)	37.0 –	37.0 –				
2	F, A, H, RE			NA + (5,191.4)	39.6§ + (3,655.2)	38.4 + (119.1)	38.0 + (1.3)	37.6 –	38.5	38.0	37.4		
3	F, A, R, H				NA + (155.5)	39.3§ + (385.2)	37.0 + (3.7)	37.2 + (0.4)	37.3 –	37.2 –			
4	F, A	NA + (0.8)		40.8§ + (14,605.3)	39.9 + (14,170.2)	38.2 + (1,007.8)	37.6 + (4.0)	37.6 + (0.2)	37.4 –	37.0			
5	F, A		NA + (371.4)		37.0§ + (25.9)	37.8 + (0.4)	36.8 –	36.6 –					
6	F, D, R, M			NA + (524.4)	37.6§ + (0.7)	38.6 + (0.4)	37.2 –	36.8 –					
7	F, A, H, EP			NA + (18.8)	38.3§ + (7.2)	37.0 + (0.4)	37.0 –						
8	F, A								NA + (36.6)	37.5§	37.6 + (0.3)	37.7 –	36.4
9	F, A, N			NA + (406.7)	38.4§ –	37.8	37.1	37.1					
10	F, A, M						39.2§	36.7 + (8.5)	36.8 + (3.6)	37.1 –	37.4 –	36.8	

*At hospitalization. F, fever; A, arthralgia; B, backache; D, diarrhea; H, headache; RE, red eyes; R, rash; M, myalgia; EP, eye pain; N, nausea. †Day 0, day of fever onset. Maximum temperature expressed in ºC. Light shading indicates self-reported fever; dark shading indicates documented fever (maximum temperature >37.5 ºC). NA, not available. ‡Viral load expressed as × 10^3^ pfu/mL. Reverse transcription–PCR test results for chikungunya: +, positive; –, negative. §Indicates day patient was hospitalized.

*Aedes aegypti* mosquitoes were the vectors involved in this outbreak. Viral sequences from our patients showed a close association to the circulating strains in the 2006 Indian Ocean outbreak (GenBank accession nos. EU441882 and EU441883), without the E1-A226V mutation, which can increase transmission of the virus in the alternate vector *Ae. albopictus* (*5*). Virus phylogenetic studies supported the notion that the East African genotype, which emerged in Kenya in 2004 and the Indian Ocean islands in 2005, closely resembling the 2006 outbreak strain in India, arrived in Singapore in January 2008.

Singapore’s outbreak containment strategy focused primarily on intensive vector control and rapid removal of infectious human reservoirs through active case finding and isolation. The proportion of asymptomatic infections in this outbreak was not determined. Asymptomatic infections could possibly reduce the effectiveness of control efforts. However, there ha ve been no data thus far supporting chikungunya transmissibility in asymptomatic persons. Detectable viremia before clinical signs and symptoms and high levels of viremia during early illness, as demonstrated in our study and others (*9**,**10*), pose logistical challenges in the timeliness of case detection for isolation.

Singapore remains at risk for chikungunya outbreaks. It has a highly susceptible population, a porous border with large travel volumes from epidemic areas, and effective vectors (both *Ae. aegypti* and *Ae. albopictus).* In the absence of a vaccine, high vigilance for autochthonous transmission and stringent vector control should be maintained along with a swift public health response.
